# Response to a DNA vaccine against the H5N1 virus depending on the chicken line and number of doses

**DOI:** 10.1186/s12985-020-01335-9

**Published:** 2020-05-07

**Authors:** Barbara Małgorzata Kalenik, Anna Góra-Sochacka, Anna Stachyra, Monika Olszewska-Tomczyk, Anna Fogtman, Róża Sawicka, Krzysztof Śmietanka, Agnieszka Sirko

**Affiliations:** 1 Institute of Biochemistry and Biophysics, Polish Academy of Sciences, Pawinskiego 5A, 02-106 Warsaw, Poland; 2 Department of Poultry Diseases, National Veterinary Research Institute, Al. Partyzantow 57, 24-100 Puławy, Poland

**Keywords:** Broilers, Chicken, DNA vaccine, Influenza, Layers, Spleen transcriptome, SPF chickens

## Abstract

**Background:**

Avian influenza virus infections cause significant economic losses on poultry farms and pose the threat of a possible pandemic outbreak. Routine vaccination of poultry against avian influenza is not recommended in Europe, however it has been ordered in some other countries, and more countries are considering use of the avian influenza vaccine as a component of their control strategy. Although a variety of such vaccines have been tested, most research has concentrated on specific antibodies and challenge experiments.

**Methods:**

We monitored the transcriptomic response to a DNA vaccine encoding hemagglutinin from the highly pathogenic H5N1 avian influenza virus in the spleens of broiler and layer chickens. Moreover, in layer chickens the response to one and two doses of the vaccine was compared.

**Results:**

All groups of birds immunized with two doses of the vaccine responded at the humoral level by producing specific anti-hemagglutinin antibodies. A response to the vaccine was also detected in the spleen transcriptomes. Differential expression of many genes encoding noncoding RNA and proteins functionally connected to the neuroendocrine-immune system was observed in different immunized groups.

**Conclusion:**

Broiler chickens showed a higher number and wider range of fold-changes in the transcriptional response than laying hens.

## Background

Avian influenza is a major zoonotic viral disease that causes significant adverse impacts on poultry production and the global trade [1]. Previous outbreaks have caused the loss of hundreds of millions of birds, and total economic losses are estimated to be far in excess of US $10 billion [2, 3]. Vaccination of poultry was implemented in many of the affected countries, especially in those where H5N1 viruses have become enzootic in poultry and wild birds [3–6]. Mandatory vaccinations of chickens with inactivated or recombined H5N1 viruses are considered to prevent disease and mortality in chickens, reduce human cases and help to maintain rural livelihoods and food security [7]. Moreover, vaccination is thought to be the most effective method to prevent influenza infection [8].

One of the most interesting approaches to influenza immunization is the use of DNA vaccines. Among the many advantages of this technique, it is worth highlighting that DNA vaccines are fast to produce and modify; foreign antigenic protein produced within the host cells can induce humoral and cellular immune responses and DNA vaccination leads to immunization with an antigen likely to be folded in its native conformation, correctly glycosylated and having normal post-translational modifications [9, 10]. This last feature is important, because the glycosylation of HA modulates among other host immune response [11]. Moreover, they are safe due to the absence of infective agents and the possibility of using a single selected antigen, which allows for the differentiation of infection in vaccinated animals [10, 12].

Many DNA vaccine candidates for protecting chickens from avian influenza have been described [10, 13, 14], and one such vaccine has been licensed for use in the U.S. [15]. Protective DNA vaccine candidates were also designed and tested by our team [16–20].

Monitoring the transcriptome of vaccinated animals can allow for the discovery of vaccine-induced correlates of protection [21, 22]. To our knowledge, there are only three articles describing the transcriptomic response of chickens vaccinated against avian influenza virus. Two of them showed the response of birds vaccinated with inactivated low-pathogenic H9N2 virus (A/Chicken/United Arab Emirates/99), with or without an adjuvant, and subsequently infected with homologous virus [23, 24]. The third one, by our group, analysed the spleen transcriptome of broilers (Ross 308) vaccinated with two doses of protein (protein/protein), two doses of DNA (DNA/DNA), or the combined prime/boost (DNA/protein) vaccine against H5 avian influenza [25].

Herein, we compare the previously reported changes in the transcriptomic profiles of the Ross 308 line vaccinated twice with DNA vaccine with the changes in the transcriptomic profiles of laying chickens of two lines, White Leghorn maintained under specific pathogen-free (SPF) conditions and Rosa 1 maintained under standard bedding conditions. Additionally, the transcriptomic profiles of Rosa 1 after one dose of DNA vaccine are presented and discussed.

## Methods

### Plasmid used for DNA vaccination

The plasmid containing the cDNA encoding full-length (except the proteolytic cleavage site between the HA1 and HA2 subunits, residues 341–347) hemagglutinin (HA) from A/swan/Poland/305-135 V08/2006 (H5N1), clade 2.2, with codons optimized for the domestic chicken was used for DNA vaccination. Codon bias was described earlier as K3 [17, 20].

### Immunization experiment and spleen collection

Rosa 1 chickens were purchased from a commercial breeder on the day of hatching and maintained at an experimental poultry house under standard bedding conditions. Specific pathogen-free (SPF) White Leghorn (WL) chickens were purchased from VALO Biomedia (Germany) and housed in a biosafety level 3 containment facility of the National Veterinary Research Institute, Pulawy. All groups of birds were primed intramuscularly with the DNA vaccine containing 60 μg of plasmid DNA complexed with Lipofectin on day 7. The Rosa [1x] group was not given a second dose of the vaccine, while the Rosa [2x] and WL [2x] groups were boosted on day 21. Animals were sacrificed 7 days after the last immunization; for Rosa [1x] on day 14 of the animal’s life, and in the case of Rosa [2x] and WL [2x], on day 28 of the animal’s life, and blood samples and spleens were collected from the experimental groups and the corresponding control groups, which were treated with empty plasmid. The spleens were immediately immersed in RNA*later* reagent (Ambion 5:1; RNA*later*:tissue; v:v). In this study we compare results of immunization of the Rosa 1 and WL breeds with previously published results of immunization of the Ross 308 breed, where the group was referred to as the DNA/DNA group [18, 25].

### Enzyme-linked immunosorbent assay (ELISA)

Indirect ELISA for detection of anti-HA antibodies in serum was performed as described previously [17].

### Hemagglutination inhibition (HI)

HI tests were performed according to the OIE standard procedures as described earlier [20]. The hemagglutinating antigen from strains A/turkey/35/07 (clade 2.2) and A/crested eagle/Belgium/H5N1/ (clade 1) (kindly provided by Dr. Thierry van den Berg, Brussels, Belgium) were used in the WL [2x] group, while the commercially available hemagglutinating antigen (with 96% protein sequence similarity to the vaccine antigen) prepared from the low pathogenic H5N2 strain A/chicken/Belgium/150/1999 was used in the Ross [2x] group. HI titres are shown as the reciprocal of the highest dilution of sera that completely inhibited hemagglutination.

### RNA isolation and microarray experiments

RNA isolation and microarray experiments were performed as described previously [25].

### Availability of data and materials

The datasets supporting the conclusions of this article are available in the GEO repository, accession number GSE135671 and GSE102972.

### Microarray data analysis

Microarray data were analysed as described previously [25]. Venn diagrams were drawn using UGent webtool (http://bioinformatics.psb.ugent.be/webtools/Venn/). Remaining plots were made with MS Excel 2007.

### Statistical analysis

Statistical analysis was performed with R Statistical Software [26]. One-factor simple analysis of variance was done for comparison of mean HI titre between WL and Ross chickens. One-factor simple analysis of variance and Tukey-HSD test was done for comparison of mean ELISA results. Two-way Pearson’s linear correlation test was done for the correlation analysis.

## Results

### Humoral response in sera of chickens used for microarray experiments

**Fig. 1:**
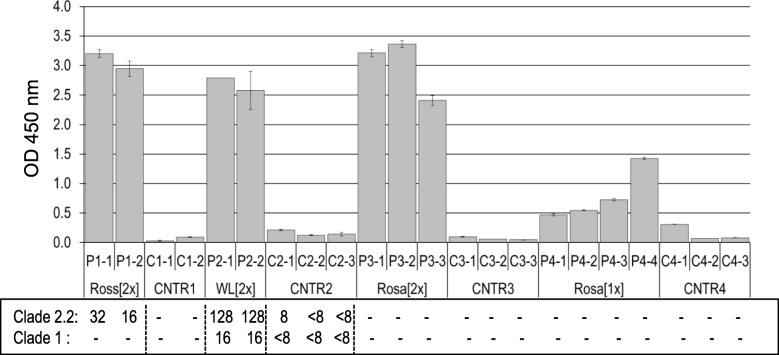
Humoral response in sera of immunized chickens used for microarray experiments. ELISA results are show in the chart, while the results of HI determination using two types of antigens are shown below, if available; the absence of an HI value means that HI was not determined in this individual. Results are shown for immunized (P) and control (C) individuals. The controls (CNTR1–4) were maintained at the same time as the respective immunized chickens, but instead of the DNA vaccine, they obtained an identical dose of the empty vector

The level of anti-H5 HA antibodies in sera collected from birds used in microarray experiments is shown in Fig. 1. All groups of boosted chickens (Ross [2x], WL [2x] and Rosa [2x]) had significantly higher levels of anti-H5 HA antibodies in their sera than birds from the Rosa [1x] group, which were given only one dose of the vaccine (*p* < 0.0001 for all groups), however differences in ELISA results between the groups of boosted chicken were not significant. As expected, the sera of control birds tested negative in ELISA. The HI titre was assayed in selected groups in the sera of vaccinated chickens (Ross [2x] and WL [2x] groups). The HI titre indicated that the used vaccine stimulated a protective response, particularly in WL chickens, which had SPF status. HI titer in the WL [2x] group was significantly higher than HI titer in the Ross [2x] group [F (1,2) = 169; *p* = 0.006]. HI titer in Rosa chickens was not determined. The maximal HI titre in control animal sera was about 8, which is generally considered background level.

We found a very strong negative correlation (*r* = − 0.994; *p*-value = 0.006; *N* = 4) between HI titer of WL [2x] and Ross [2x] chickens and the DEGs number in these groups. The same correlation was observed between HI titer of WL [2x] and Ross [2x] chickens and the Fold-Change range. There was no significant correlation between ELISA results and the number of DEGs nor between ELISA results and Fold-Change range.

### Overview of differentially regulated transcript clusters and genes

**Fig. 2:**
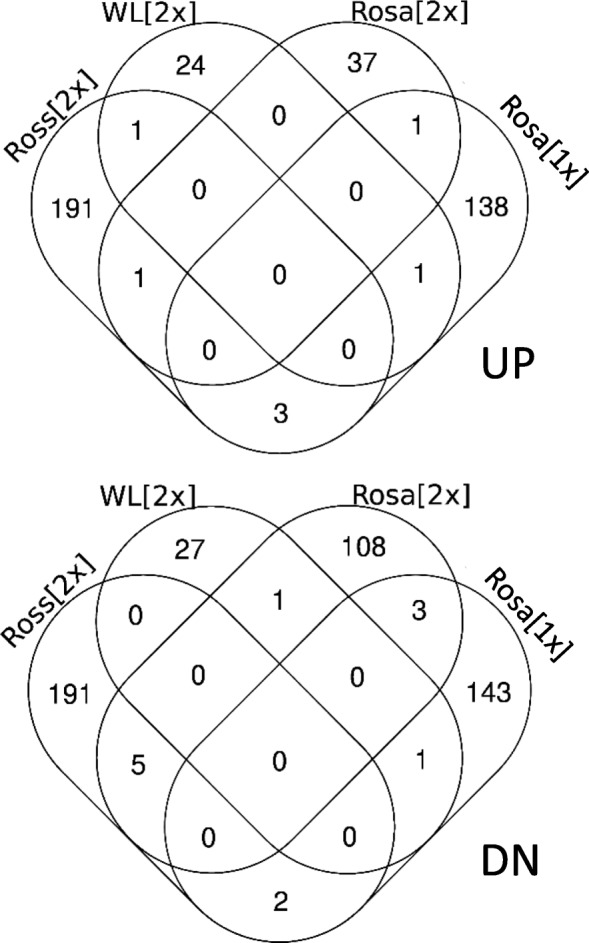
Venn diagrams of differentially expressed transcript clusters in the immunized groups

The lists of transcript clusters which were up- or downregulated (min ± 1.3-fold) with *p* ≤ 0.05 in chickens from the respective vaccination variants in comparison with the appropriate control groups are shown in Tables S1-S4. A total of 394 (188 up- and 196 downregulated), 55 (29 up- and 26 downregulated), 156 (117 up- and 39 downregulated) and 292 (149 up- and 143 downregulated) transcript clusters showed differential expression in the Ross [2x], WL [2x], Rosa [2x] and Rosa [1x] groups, respectively, compared with the unvaccinated controls (Fig. 2). Most (98.7%) of these transcript clusters were annotated by Affymetrix or successfully identified using BLAST. In the microarray chips we used, a single gene may be represented by more than one transcript cluster; therefore, the number of identified differentially expressed genes (DEGs) has been reduced to 375 and 279 in the Ross [2x] and Rosa [1x] groups, respectively, while in the WL [2x] and Rosa [2x] groups the number of DEGs was equal to the number of differentially expressed transcript clusters. Most DEGs were unique to a single immunized group; however, 34 DEGs were common to any two immunized groups (Table S5). About 56% of these DEGs were regulated in the same direction in both groups, of which 12 were upregulated and 7 were downregulated. One of the most strongly upregulated genes common to the Ross [2x] group (6.3-fold) and the Rosa [2x] group (2.3-fold) was GVINP1 (GTPase, very large interferon inducible pseudogene 1).

### Functional analysis of DEGs

Significantly enriched GO Terms (*p* < 0.05) for the Ross [2x], Rosa [2x] and Rosa [1x] groups are listed in Table 1. No significantly enriched GO Terms were identified in the WL [2x] group. All GO Terms were unique; however, positive regulation of mast cell degranulation (BP:GO:0043306), significantly enriched in the Ross [2x] group, is clearly related to the immune response enriched in the Rosa [2x] group.

**Table 1:** Top GO terms that were significantly enhanced in the vaccinated groups. BP, Biological Process, MF, Molecular Function

GO Category ID	Description	Gene count	***p***-value
**Ross [2x]**			
BP: GO:0043306	positive regulation of mast cell degranulation	2	2.8E-02
BP: GO:0051091	positive regulation of sequence-specific DNA binding transcription factor activity	4	1.5E−2
MF: GO:1990782	protein tyrosine kinase binding	2	2.6E-2
BP: GO:0030154	cell differentiation	6	2.8E-2
BP: GO:0003151	outflow tract morphogenesis	3	3.5E-2
BP: GO:0007223	Wnt signalling pathway, calcium modulating pathway	2	3.5E-2
BP: GO:0034446	substrate adhesion-dependent cell spreading	3	4.0E-2
BP: GO:0090090	negative regulation of canonical Wnt signalling pathway	4	4.40E-3
**Rosa [2x]**			
MF: GO:0004060	arylamine N-acetyltransferase activity	2	3.0E-2
BP: GO:0006955	immune response	4	4.9E-2
**Rosa [1x]**			
BP: GO:0007154	cell communication	4	6.7E-4
BP: GO:0032781	positive regulation of ATPase activity	3	6.3E-3
BP: GO:0006629	lipid metabolic process	4	1.4E-2
BP: GO:0001666	response to hypoxia	4	2.1E-2
BP: GO:0051592	response to calcium ion	3	2.9E-2
BP: GO:0086005	ventricular cardiac muscle cell action potential	2	4.4E-2

**Fig. 3:**
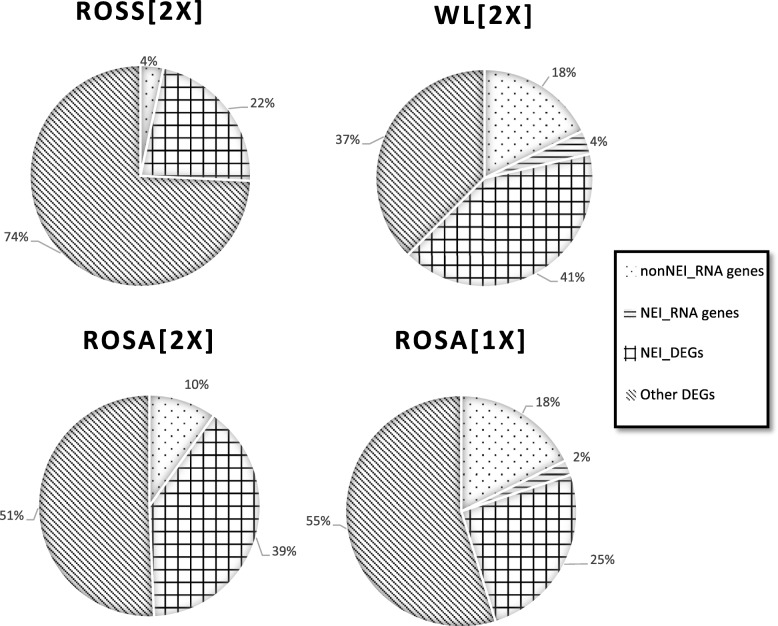
Enriched groups of DEGs among all DEGs identified in the study. NEI_RNA genes, DEGs encoding RNA related to the neuroendocrine-immune system; nonNEI_RNA genes, DEGs encoding RNA without known relation to the neuroendocrine-immune system; NEI_DEGs, DEGs encoding proteins related to the neuroendocrine-immune system; OtherDEGs DEGs encoding proteins without known relation to the neuroendocrine-immune system

Further analysis of individual DEGs allowed us to distinguish two general subsets: (i) RNA-encoding DEGs (Table S6) and (ii) DEGs connected to neuroendocrine-immune system (NE_Imm DEGs, Table S7). Representation of these categories was varied in different immunized groups (Fig. 3). The RNA-encoding DEGs were most frequently represented in the WL [2x] group (22% of all DEGs) and in the Rosa [1x] group (20% of all DEGs), while in the Rosa [2x] and the Ross [2x] groups, they represented only 10 and 4% of all DEGs, respectively. The NE_Imm DEGs were most frequently represented in the WL [2x] group (50% of all DEGs) and in the Rosa [2x] group (43% of all DEGs).

### RNA-encoding DEGs

**Fig. 4:**
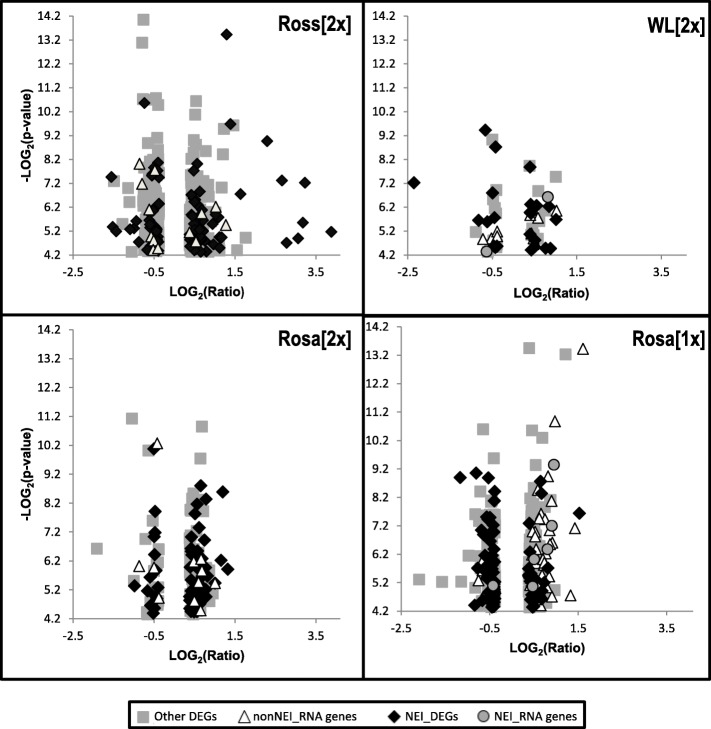
Scatterplots highlighting regulation of enriched groups of DEGs among all DEGs identified in the study. NEI_RNA genes, DEGs encoding RNA related to the neuroendocrine-immune system; nonNEI_RNA genes, DEGs encoding RNA without known relation to the neuroendocrine-immune system; NEI_DEGs, DEGs encoding proteins related to the neuroendocrine-immune system; OtherDEGs DEGs encoding proteins without known relation to the neuroendocrine-immune system

Most of the RNA-encoding DEGs (13, 10, 10 and 43 in the Ross [2x], WL [2x], Rosa [2x] and Rosa [1x] groups, respectively) belonged to the miRNA and snoRNA classes. SNORA56 was differentially expressed in two groups, WL [2x] and Rosa [1x] (Table S5); however, it was downregulated in the former and upregulated in latter. Differences in the expression of this gene seem to be quite high (fold-change: − 1.65 vs. 3.05). Other snoRNAs that were differentially expressed in more than one group included: (i) SNORA5 and SNORD35, which were upregulated in the WL [2x] and Rosa [1x] groups; (ii) SNORA26, which was upregulated in the Rosa [2x] and Rosa [1x] groups and (iii) SNORA74 and MIR1757, which were differentially regulated in the Ross [2x] and Rosa [1x] groups. Interestingly, the former was down- and upregulated, whereas the latter was up- and downregulated in the Ross [2x] and Rosa [1x] groups, respectively. Interestingly, the genes from this category were highly overrepresented among the 15 most upregulated genes in the Rosa (1x) group (Fig. 4, Table 2). Two and six of the miRNAs encoding DEGs from the WL [2x] and Rosa [1x] groups, respectively, have been reported in connection with the neuroendocrine-immune system (Table S7).

**Table 2:** TOP 15 up- and downregulated DEGs in the vaccinated groups. RNA-encoding DEGs and DEGs connected to the neuroendocrine-immune system are bolded and underlined, respectively

Ross[2x]		WL[2x]		Rosa[2x]		Rosa[1x]	
Up-regulated							
Gene Symbol	FC	Gene Symbol	FC.	Gene Symbol	FC	Gene Symbol	FC
GAL7	14,7	***SNORD35***	2,03	MX1	2,50	***SNORA56***	3,05
GAL1	9,4	GRM8	2,02	GVIN1	2,30	MHC region	2,88
GAL2	9,1	USP6NL	2,00	ISG12–2	2,23	***U11***	2,68
GAL6	8,3	MGST2	1,84	MHC	2,01	***MIR1743***	2,51
LECT2	6,8	MHC class I	1,80	***ncRNA***	2,01	–	2,32
GVIN1	6,3	***gga-mir-147***	1,76	***ncRNA***	2,01	***MIR1596***	1,96
TCRDV	4,9	OAT	1,69	***ncRNA***	2,01	C9ORF58	1,96
6TBGa-2	3,4	SLC2A9	1,68	***ncRNA***	2,01	***MIR7B***	1,93
SERPINB10	3,1	C2H6ORF105	1,53	LAMA1	1,93	***SNORA66***	1,89
LOC100858620	2,9	BORCS5	1,50	ZP1	1,83	***SNORA23***	1,87
RHCE	2,8	***NONGGAT007307***	1,50	TSPO2	1,82	***MIR1680***	1,87
S100A9	2,6	NADB-LER2	1,49	LAG3	1,77	***SNORD90***	1,87
ACKR2	2,5	STAM2	1,47	ARHGEF39	1,73	RPL23	1,84
***SNORD24***	2,4	TIFA	1,42	CCR8	1,72	***SNORD14***	1,82
TIMMDC1	2,3	CCLi9 gene	1,42	LYGL	1,72	ND4L	1,81
Down-regulated							
DDX60	-2,9	IGLV	−5,12	–	−3,77	PRED.: LOC107050276	−4,28
DDX60	−2,9	GDPD2	−1,85	–	−2,07	CIAO1	−2,98
***snoRNA GGN47***	−2,9	LRRC4C	−1,77	GIMAP7L5	−2,00	SLC8A3	−2,25
NTMT1	−2,8	***SNORA56***	−1,65	IL28RA	−1,97	PRED.: MICA	−2,21
EYA1	−2,7	IFNA	−1,58	***MIR1637***	−1,82	PRED.: CYP2J2L2	− 1,97
RRBP1	−2,4	***MIR1636***	−1,55	TDRD5	−1,64	CHIR-B5	−1,79
GPR158	−2,2	AICDA	−1,53	GRTP1	−1,60	LOC431321	−1,78
LRRC3B	−2,2	ACTA2	−1,52	MRPL13	−1,59	PRED.: LOC100859143	−1,77
ALDH1A3	−2,1	LOC100858370	−1,46	CACNB4	−1,58	PTPRZ1	−1,75
STXBP5L	−2,1	***MIR1648***	−1,43	ABI3BP	−1,56	COCH	−1,70
IFIT5	−2,0	ACTG2	−1,41	VH57–1	−1,51	***Mt_tRNA***	−1,69
OASL	−1,9	SLC8A3	−1,41	LOC107050168	−1,51	PRED.: TMEM98	−1,67
MDK	−1,8	LOC776492	−1,40	LACC1	−1,50	LECT1	−1,65
***MI0005509***	−1,8	OLFML3	−1,39	LOC107055505	−1,45	PRED.: OR14A16L45	−1,64
***SNORD18***	−1,7	TMC6	−1,37	KCNK12	−1,45	SLC38A3	−1,63

### DEGs related to the neuroendocrine-immune system

This category included 86, 23, 60 and 74 DEGs in the Ross [2x], WL [2x], Rosa [2x] and Rosa [1x] groups, respectively (Table S7). Many of them were present only in one vaccinated group. Others, like the genes encoding the major histocompatibility complex (MHC), form a very large set, with members present in different groups, and most of them were upregulated. The remaining genes from this category that were present in more than one group included genes encoding: (i) Nestin (NES), which was downregulated in the Ross [2x], WL [2x] and Rosa [1x] groups, (ii) Solute Carrier Family 8 Member A3 (SLC8A3), which was downregulated in the WL [2x] and Rosa [1x] groups, (iii) Potassium Calcium-Activated Channel Subfamily M Alpha 1 (KCNMA1), which was upregulated in the Rosa [2x] and Rosa [1x]) groups, (iv) Argininosuccinate synthase 1 (ASS1), encoding protein engaged in negative regulation of leukocyte cell-cell adhesion, which was down- and upregulated in the Rosa [2x] and Rosa [1x] groups, respectively, (v) heat shock protein HSPB1, which was downregulated in the Ross [2x] and Rosa [1x] groups, (vi) Granzyme M (GZMM) and Colony stimulating factor 3 receptor (CSF3R), both upregulated in the Ross [2x] and Rosa [2x] groups, (vii) Calpain 2 (CAPN2) which was down- and upregulated in the Ross [2x] and Rosa [2x] groups, respectively and (viii) EF-hand calcium-binding domain-containing protein 4B (EFCAB4B), which was up- and downregulated in the Ross [2x] and Rosa [2x] groups, respectively.

The DEGs from this category were highly overrepresented among the 15 most upregulated genes in the Ross [2x]) group and also (but to a much lesser extent) in the WL [2x] group (Table 2, Fig. 4). Moreover, the NE_Imm DEGs were the only DEGs with a log_2_(ratio) higher than 2 (Fig. 4).

## Discussion

The effects of immunization were first verified at the humoral level. All groups of birds immunized with two doses of the vaccine responded by producing specific anti-HA antibodies. Then, we focused on changes in the spleen transcriptome, evaluated in each immunized group in relation to their respective controls. Immunization of broiler chickens (the Ross [2x] group) resulted in the largest number of DEGs, while vaccination of White Leghorn SPF chickens (the WL [2x] group) revealed the smallest number of DEGs (Fig. 2, Fig. 4). Interestingly, despite a low level of anti-H5 HA antibodies in sera, the Rosa [1x] group, immunized with one dose, showed more DEGs and higher fold-changes than the Rosa [2x] group, vaccinated twice (Fig. 1, Fig. 2, Fig. 4). All chickens vaccinated twice showed similar level of anti-H5 HA antibodies in sera (Fig. 1), however White Leghorn SPF chickens displayed significantly higher HI titre than Ross chickens (and HI titre of Rosa chickens was not determined). HI titre was negatively correlated with the number and Fold-Change range of DEGs. We believe that strong and quick secondary response initiated by memory cells can cause lower changes in gene expression in spleen at day 7 post vaccination.

Moreover, the microarray chips used in this work (Affymetrics Chicken Gene 1.1 ST Array) were built, according to the Affymetrix DataSheet, on the galGal3 genome founded on the Red Jungle fowl (*Gallus gallus*), one of the main ancestors of domestic chickens (*Gallus domesticus)*. Some studies imply that broiler chickens are more closely related to Red Jungle fowl than layers [27], which could be the reason for the higher number of DEGs detected in broilers (the Ross [2x] group) than in the layers represented by all remaining groups (Fig. 2). On the other hand, broilers may also show more DEGs because of breed selection. For example, the transcriptional profile of the breast muscle in heat-stressed layers was similar to that of broiler chickens kept at the control temperature, while heat stress amplified changes in broilers [28].

The chicken genome was the first completed genome of a breeding animal [29]; now, the fourth version (Gallus_gallus-5.0), corrected among others by annotation of 2768 noncoding genes and many CHIR loci, has been released [30]. The number of chicken mRNAs seems to be lower than in humans [31]; however, the chicken transcriptome seems to show a similar level of complexity [32]. That explains the relatively high representation of RNA-encoding DEGs in our results. Moreover, mammalian long non-coding RNAs (lncRNAs) were reported to play critical roles in the immune response to influenza A virus infection [33], and some lncRNAs were identified as being related to the immune response to influenza A virus in ducks [34]; however, the roles of many non-coding RNAs remain to be discovered. Interestingly, the proportion of RNA-encoding DEGs of all DEGs is far less in broilers (the Ross [2x] group) than in all remaining groups (Fig. 3). In fact, the number of RNA-encoding DEGs in both groups of Rosa 1 chickens (Rosa [2x] and Rosa [1x]) is higher than that in broilers (the Ross [2x] group), despite a significantly smaller overall number of DEGs (Fig. 2, Fig. 4, Table S6). The RNA-encoding DEGs reported in this study belong mainly to the miRNA and snoRNA classes (Table S6). Deep sequencing of the transcriptomes of skeletal muscles from broiler and layer chickens showed that they share a few millions common miRNAs; however, tens of thousands miRNAs were still specific to either broiler or layer skeletal muscle [35]. Interestingly, during that study the sequence tag annotations demonstrated that known chicken miRNAs and metazoan miRNA homologs accounted for about 50% of all sequence reads in the broiler and layer libraries, whereas snoRNAs were only slightly represented, although sequencing was in that case performed by fractionating total RNA using polyacrylamide gel electrophoresis to enrich for molecules in the range of 16–30 nt [35]. Differential expression of many miRNAs between the lungs of broilers infected with H5N3 and those of non-infected animals was also reported previously [36]. Some snoRNAs were differentially expressed between the various immunized groups (Table S5). Despite the variety in chicken breeds and differences in maintenance conditions, some of these differences may be caused by the recognition of snoRNA derivatives by the Transcript Clusters optimized to detect snoRNA molecules [37, 38]. Moreover, Transcript Clusters optimized for detection of certain mRNA can detect also regulatory RNA made from pseudogenes [39]. This fact, together with different time points, can explain differences between expression level of some DEGs (e.g. ASS1, EFCAB4B and UTS2R) in various studied group.

Our study reports differential regulation of many ImmDEGs in spleens of chickens vaccinated with the experimental DNA vaccine (Table S7). Among them one can find numerous DEGs encoding cytokines (CCLi9, IL5 and IL17D) and their receptors (CCR7, CCR8, CCR8L, CX3CR1, CXCR4, IL1R2, IL5RA, IL12RB2, IL17REL and IL28RA). To our knowledge, at least 10 of the ImmDEGs identified in this study (TLR2–1, IRG1, MX1, OASL, IFIT5, CXCR4, DDX60, NFKBIZ, IFNA and IL12RB2) were reported as differentially expressed during experimental infection of chickens or chicken cells with H5 influenza viruses. TLR2–1 was overexpressed in the lungs of chickens infected with the highly pathogenic AIV H5N1 strain A/Chicken/Jiangsu/k0402/2010 [40] and was found by us as a DEG in the WL [2x] group. IRG1 was downregulated in CEF cells infected with H5N2 virus [41] and was identified by us as a DEG in the Rosa [1x] group. The regulation of these two genes observed in this study seems to have proinflammatory consequences (Table S7).

MX1, OASL and IFIT5 genes encode proteins with known functions in influenza defense [41]. MX1 was the most overexpressed gene in the Rosa 1 [2x] group (Table 2), and its overexpression was also reported in CEF cells and chickens infected with H5N1 or H5N2 viruses [40, 42] and in the lungs of chickens infected with H5N1 virus from 24 h post infection [43]. The OASL gene was downregulated in the Ross [2x] group; however, it was upregulated in CEF cells infected with H5 viruses at 4 h post infection [42]. Regulation of the expression of this gene in the lungs of chickens infected with highly pathogenic H5N1 virus depended on the time point [40]. IFIT5 was overexpressed in CEF cells infected with H5 viruses at 12 h post infection [40] and downregulated in the Ross [2x] group. Differences in expression of the OASL and IFIT5 genes may result from differences in the experimental setup. Regulation of CXCR4, DDX60 and NFKBIZ varied at different time points after infection of chicken lungs [40]. IFNA was up- and IL12RB2 was downregulated in lungs of chickens infected with H5N1 virus [43], whereas they were upregulated in the spleens of chickens vaccinated in this study (the WL [2x] and Ross [2x] group, respectively).

In summary, broiler chickens (Ross 308) showed a higher number and wider range of fold-changes in the transcriptional response than laying hens (White Leghorn or Rosa 1). Interestingly, White Leghorn SPF chickens had a lower number and lower range of fold-changes than the Rosa 1 breed. Moreover, the number and range of gene expression changes was higher in the Rosa 1 group that received one dose than in the Rosa 1 group that was boosted. In all groups many RNA-encoding DEGs and DEGs connected to the neuroendocrine-immune system were identified. Their representation was higher in laying chicken breeds than in broilers. Some genes (detected in this study) functionally connected to the immune response were also reported as differentially expressed during experimental influenza infection of chickens or chicken cells.

## Conclusion

Our results indicate that different chicken breeds might respond differentially to the vaccination. The vaccination stimulates response in spleen transcriptome which could be used in future for selection of the markers of the vaccine effectiveness.

## Supplementary information

**Additional file 1 **Tables S1-S7. **Table S1.** Complete list of genes differentially regulated in chickens from the Ross [2x] group in comparison with chickens from the corresponding control group. **Table S2.** Complete list of genes differentially regulated in WL [2x] chickens in comparison with chickens from the respective control group. **Table S3.** Complete list of genes differentially regulated in chickens from the Rosa [2x] group in comparison with chickens from the respective control group. **Table S4.** Complete list of genes differentially regulated in chickens from the Rosa [1x] group in comparison with chickens from the respective control group. **Table S5.** Differentially expressed genes present in at least two groups of immunized chickens. **Table S6.** List of RNA-encoding DEGs. ‘U’ and ‘D’ means that indicated sequence was recognized as up- and down-regulated, respectively. **Table S7.** List of DEGs connected to the neuroendocrine-immune system. ‘Imm’, ‘N’ and ‘E’ in ‘System part’ column indicate function of the gene connected to the immune, neurological and endocrinological part of neuroendocrine-immune system, respectively.

## Data Availability

The data discussed in this publication (accession number GSE135671 and GSE102972) are accessible through GEO Series (https://www.ncbi.nlm.nih.gov/geo).

## Abbreviations

AIV: Avian influenza virus
DEGs: Differentially expressed genes
HA: Hemagglutinin
ImmDEGs: DEGs functionally connected with the immune system
NE_Imm DEGs: DEGs connected to the neuroendocrine-immune system

## References

[1] Jang H, Elaish M, Kc M, Abundo MC, Ghorbani A, Ngunjiri JM, Lee CW (2018). Efficacy and synergy of live-attenuated and inactivated influenza vaccines in young chickens. PLoS One.

[2] Chmielewski R, Swayne DE (2011). Avian influenza: public health and food safety concerns. Annu Rev Food Sci Technol.

[3] Yoo SJ, Kwon T, Lyoo YS (2018). Challenges of influenza a viruses in humans and animals and current animal vaccines as an effective control measure. Clin Exp Vaccine Res.

[4] Hasan NH, Ignjatovic J, Peaston A, Hemmatzadeh F (2016). Avian influenza virus and DIVA strategies. Viral Immunol.

[5] Li C, Bu Z, Chen H (2014). Avian influenza vaccines against H5N1 'bird flu'. Trends Biotechnol.

[6] Swayne DE, Spackman E (2013). Current status and future needs in diagnostics and vaccines for high pathogenicity avian influenza. Dev Biol (Basel).

[7] Swayne DE (2012). Impact of vaccines and vaccination on global control of avian influenza. Avian Dis.

[8] Soema PC, Kompier R, Amorij JP, Kersten GF (2015). Current and next generation influenza vaccines: formulation and production strategies. Eur J Pharm Biopharm.

[9] Shedlock DJ, Weiner DB (2000). DNA vaccination: antigen presentation and the induction of immunity. J Leukoc Biol.

[10] Stachyra A, Gora-Sochacka A, Sirko A (2014). DNA vaccines against influenza. Acta Biochim Pol.

[11] Wu CY, Lin CW, Tsai TI, Lee CD, Chuang HY, Chen JB, Tsai MH, Chen BR, Lo PW, Liu CP (2017). Influenza a surface glycosylation and vaccine design. Proc Natl Acad Sci U S A.

[12] Uttenthal A, Parida S, Rasmussen TB, Paton DJ, Haas B, Dundon WG (2010). Strategies for differentiating infection in vaccinated animals (DIVA) for foot-and-mouth disease, classical swine fever and avian influenza. Expert Rev Vaccines.

[13] Meunier M, Chemaly M, Dory D (2016). DNA vaccination of poultry: the current status in 2015. Vaccine.

[14] Shan S, Fenwick S, Ellis T, Poinern E, Edwards J, Le X, Jiang Z (2016). Evaluation of different chemical adjuvants on an avian influenza H6 DNA vaccine in chickens. Avian Pathol.

[15] AgriLabs. First DNA vaccine licensed for chickens. PR newswire: Cision; 2017. https://www.prnewswire.com/news-releases/first-dna-vaccine-licensed-for-chickens-300554855.html. Accessed 28 Apr 2020.

[16] Stachyra A, Gora-Sochacka A, Radomski JP, Sirko A (2019). Sequential DNA immunization of chickens with bivalent heterologous vaccines induce highly reactive and cross-specific antibodies against influenza hemagglutinin. Poult Sci.

[17] Stachyra A, Gora-Sochacka A, Sawicka R, Florys K, Sączynska V, Olszewska M, Pikuła A, Śmietanka K, Minta Z, Szewczyk B (2014). Highly immunogenic prime–boost DNA vaccination protects chickens against challenge with homologous and heterologous H5N1 virus. Trials Vaccinology.

[18] Stachyra A, Pietrzak M, Maciola A, Protasiuk A, Olszewska M, Smietanka K, Minta Z, Gora-Sochacka A, Kopera E, Sirko A (2017). A prime/boost vaccination with HA DNA and Pichia-produced HA protein elicits a strong humoral response in chickens against H5N1. Virus Res.

[19] Stachyra A, Rak M, Redkiewicz P, Madeja Z, Gawarecka K, Chojnacki T, Swiezewska E, Masnyk M, Chmielewski M, Sirko A, Gora-Sochacka A (2017). Effective usage of cationic derivatives of polyprenols as carriers of DNA vaccines against influenza virus. Virol J.

[20] Stachyra A, Redkiewicz P, Kosson P, Protasiuk A, Gora-Sochacka A, Kudla G, Sirko A (2016). Codon optimization of antigen coding sequences improves the immune potential of DNA vaccines against avian influenza virus H5N1 in mice and chickens. Virol J.

[21] Barton AJ, Hill J, Pollard AJ, Blohmke CJ (2017). Transcriptomics in human challenge models. Front Immunol.

[22] Wang IM, Bett AJ, Cristescu R, Loboda A, ter Meulen J (2012). Transcriptional profiling of vaccine-induced immune responses in humans and non-human primates. Microb Biotechnol.

[23] Degen WG, Smith J, Simmelink B, Glass EJ, Burt DW, Schijns VE (2006). Molecular immunophenotyping of lungs and spleens in naive and vaccinated chickens early after pulmonary avian influenza a (H9N2) virus infection. Vaccine.

[24] Reemers SS, Jansen C, Koerkamp MJ, van Haarlem D, van de Haar P, Degen WG, van Eden W, Vervelde L (2010). Reduced immune reaction prevents immunopathology after challenge with avian influenza virus: a transcriptomics analysis of adjuvanted vaccines. Vaccine.

[25] Kalenik BM, Gora-Sochacka A, Stachyra A, Pietrzak M, Kopera E, Fogtman A, Sirko A (2018). Transcriptional response to a prime/boost vaccination of chickens with three vaccine variants based on HA DNA and Pichia-produced HA protein. Dev Comp Immunol.

[26] R Core Team (2020). R: a language and environment for statistical computing.

[27] Tadano R, Kinoshita K, Mizutani M, Tsudzuki M (2014). Comparison of microsatellite variations between red Junglefowl and a commercial chicken gene pool. Poult Sci.

[28] Zahoor I, de Koning DJ, Hocking PM (2017). Transcriptional profile of breast muscle in heat stressed layers is similar to that of broiler chickens at control temperature. Genet Sel Evol.

[29] Burt DW (2006). The chicken genome. Genome Dyn.

[30] Warren WC, Hillier LW, Tomlinson C, Minx P, Kremitzki M, Graves T, Markovic C, Bouk N, Pruitt KD, Thibaud-Nissen F (2017). A New Chicken Genome Assembly Provides Insight into Avian Genome Structure. G3 (Bethesda).

[31] Pertea M, Salzberg SL (2010). Between a chicken and a grape: estimating the number of human genes. Genome Biol.

[32] Kuo RI, Tseng E, Eory L, Paton IR, Archibald AL, Burt DW (2017). Normalized long read RNA sequencing in chicken reveals transcriptome complexity similar to human. BMC Genomics.

[33] Hu J, Hu Z, Wang X, Gu M, Gao Z, Liang Y, Ma C, Liu X, Hu S, Chen S (2018). Deep sequencing of the mouse lung transcriptome reveals distinct long non-coding RNAs expression associated with the high virulence of H5N1 avian influenza virus in mice. Virulence.

[34] Lu C, Xing Y, Cai H, Shi Y, Liu J, Huang Y (2019). Identification and analysis of long non-coding RNAs in response to H5N1 influenza viruses in duck (Anas platyrhynchos). BMC Genomics.

[35] Li T, Wu R, Zhang Y, Zhu D (2011). A systematic analysis of the skeletal muscle miRNA transcriptome of chicken varieties with divergent skeletal muscle growth identifies novel miRNAs and differentially expressed miRNAs. BMC Genomics.

[36] Wang Y, Brahmakshatriya V, Lupiani B, Reddy SM, Soibam B, Benham AL, Gunaratne P, Liu HC, Trakooljul N, Ing N (2012). Integrated analysis of microRNA expression and mRNA transcriptome in lungs of avian influenza virus infected broilers. BMC Genomics.

[37] Mleczko AM, Bakowska-Zywicka K (2016). When small RNAs become smaller: emerging functions of snoRNAs and their derivatives. Acta Biochim Pol.

[38] Swiatowy W, Jagodzinski PP (2018). Molecules derived from tRNA and snoRNA: entering the degradome pool. Biomed Pharmacother.

[39] Michael J, Milligan MJ, Lipovich L (2014). Pseudogene-derived lncRNAs: emerging regulators of gene expression. Front Genet.

[40] Hu J, Mo Y, Wang X, Gu M, Hu Z, Zhong L, Wu Q, Hao X, Hu S, Liu W (2015). PA-X decreases the pathogenicity of highly pathogenic H5N1 influenza a virus in avian species by inhibiting virus replication and host response. J Virol.

[41] Barber MR, Aldridge JR, Fleming-Canepa X, Wang YD, Webster RG, Magor KE (2013). Identification of avian RIG-I responsive genes during influenza infection. Mol Immunol.

[42] Sutejo R, Yeo DS, Myaing MZ, Hui C, Xia J, Ko D, Cheung PC, Tan BH, Sugrue RJ (2012). Activation of type I and III interferon signalling pathways occurs in lung epithelial cells infected with low pathogenic avian influenza viruses. PLoS One.

[43] Ranaware PB, Mishra A, Vijayakumar P, Gandhale PN, Kumar H, Kulkarni DD, Raut AA (2016). Genome wide host gene expression analysis in chicken lungs infected with avian influenza viruses. PLoS One.

